# Analytical method validation for assay determination of cannabidiol and tetrahydrocannabinol in hemp oil infused products by RP-HPLC

**DOI:** 10.1038/s41598-022-13737-6

**Published:** 2022-07-21

**Authors:** Sandhyarani Analakkattillam, Victor K. Langsi, John P. Hanrahan, Eric Moore

**Affiliations:** 1 Glantreo Limited, ERI Building, Lee Road, Cork, T23 XE10 Ireland; 2 School of Chemistry, University College Cork, Cork, T12 YN60 Ireland

**Keywords:** Health care, Medical research, Chemistry

## Abstract

A simple quantitative reverse phase high performance liquid chromatographic (RP-HPLC) method has been developed and validated for assay determination of cannabidiol and tetrahydrocannabinol in hemp oil infused products. The RP-HPLC method was developed and optimized for the mobile phase composition, flow rate, column selection and detector wavelength. An isocratic elution of samples were performed on SOLAS 100 Å C18 150 mm × 4.6 mm, 5 μm column with a mobile phase containing 75/25 acetonitrile/water v/v, with a flow rate of 1.5 mL/min by using an ultraviolet–visible (UV/Vis) detector operating at 214 nm. The RP-HPLC method was validated to meet regulatory requirements which covers specificity, accuracy, range, linearity, precision, system suitability and robustness. The validated assay test method was applied successfully to quantify cannabidiol and tetrahydrocannabinol in commercial hemp oil infused products such as tablets, soft gel capsules, plant extract oils, oral drops, tincture, and beverage enhancers. All the test results were found acceptable as per ICH guidelines, and this confirmed the feasibility of this method for its intended use in regular quality control and assay of cannabidiol and tetrahydrocannabinol in hemp oil infused products.

## Introduction

**Figure 1:**
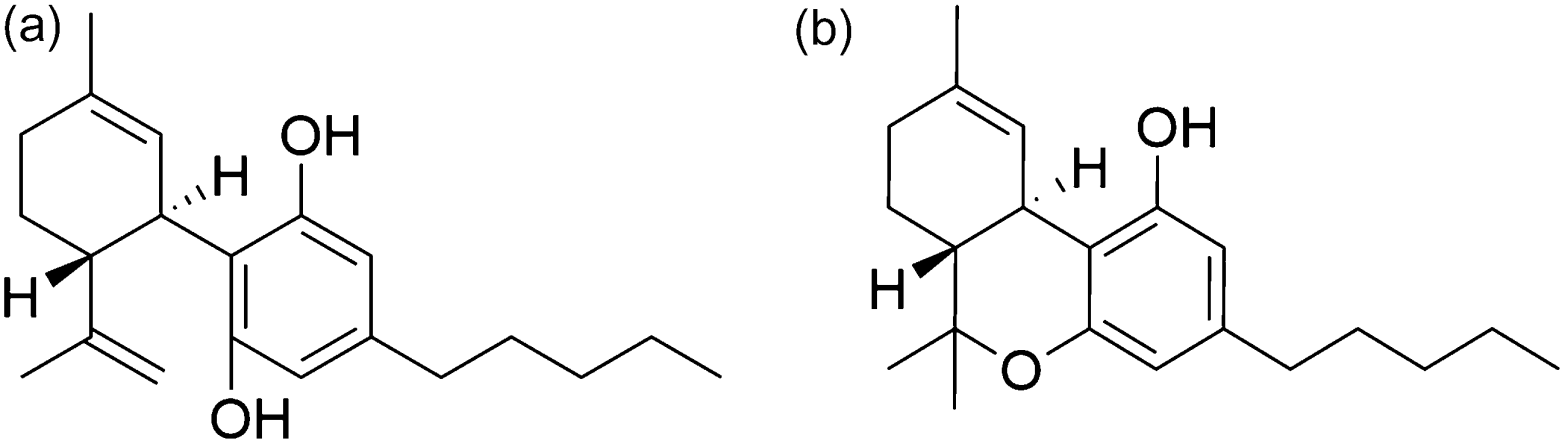
(**a**) Cannabidiol and (**b**) Tetrahydrocannabinol—structural representation of CBD and THC. Formula: C_21_H_30_O_2_; Molecular weight: 314.464 g/mol.

As the market for hemp infused products grows consumers are becoming more aware of the quality standards to which these products are tested to. Moreover, for medicinal cannabis, accurate testing is important as the patients demand specific therapeutic effects. It is for this reason that the Hemp/cannabis industry is striving for more and more actuate testing for quantifying the strength and purity of these products. Cannabis is a plant belongs to the family *Cannabaceae* and contains various natural constituents. A total of 565 compounds have been isolated and identified from the *C. sativa* species where 120 belong to the category of cannabinoids^1,2^. Cannabinoids belongs to a group of terpenophenolic compounds with 21 carbon atoms and are further divided into 11 subgroups according to their chemical structure. This constitutes 7 compounds of Cannabidiol (CBD), 16 compounds of Cannabigerol (CBG), 23 compounds of ∆9-tetrahydrocannabinol (∆9-THC), 5 compound of ∆8-tetrahydrocannabinol (∆8-THC), 11 compounds of Cannabinol (CBN), 9 compounds of Cannabichromene (CBC), 5 compounds of Cannabielsoin (CBE), 2 compounds of Cannabinodiol (CBND), 3 compounds of Cannabicyclol (CBL), 9 compounds of Cannabitriol (CBT) types and remaining 30 compounds belongs to miscellaneous type^3^. CBD, THC, CBG, CBN, and CBC are neutral cannabinoids and are synthesised by decarboxylation reaction of their respective naturally occurring acid forms (CBDA, THCA, CBGA, CBNA, and CBCA). Out of 120 cannabinoids, CBD and THC are the two major biomarkers in commercial hemp oil samples^4^. CBD (Fig. 1a), the active pharmaceutical ingredient (API) found in the medicinal hemp oil products and it shows several curative properties such as anti-psychotic, anti-anxiety, antiemetic, appetite-enhancing, analgesic, as well as muscle relaxant effects^5^. On the other hand, THC (Fig. 1b) is the principal psychoactive constituent of cannabis, and is the reason why cannabis is used as a recreational drug^5^. According to the US Farm bill 2018, hemp is defined as ‘the plant *Cannabis sativa* L. and any part of that plant, including the seeds thereof and all derivatives, extracts, cannabinoids, isomers, acids, salts, and salts of isomers, whether growing or not, with a delta-9 tetrahydrocannabinol concentration of not more than 0.3 percent on a dry weight basis’^6^. This makes the need for a quantitative method for the simultaneous determination of both CBD and THC in hemp products vitally important. The composition of both CBD and THC levels have a strong influence on the legality and physiological effects of the final product.

An extensive literature survey revealed that there have been several analytical methods to quantify cannabinoids in cannabis plant extracts and most commonly used methods utilise gas chromatography (GC) coupled with mass spectrometry (MS) detectors^7–14^. The GC method limits the quantification of acidic analogues as a result of decarboxylation due to hot inlet and oven conditions. Furthermore, the decarboxylation process was found to be incomplete and generate difficulties in quantitative analysis. In GC, the determination of acidic cannabinoids requires extensive derivatization steps^15^ which can be time consuming and costly to commercial laboratories. For the quantification of both acidic and neutral cannabinoids, methods based on high performance liquid chromatography (HPLC)^16^, thin layer chromatography (TLC)^17^, HPLC coupled with mass spectrometry (HPLC/MS)^18^, ultra-high performance supercritical fluid chromatography (UHPSFC)^19^, liquid chromatography coupled with MS (LC–MS)^20–23^, and centrifugal partition chromatographic techniques^24^ have been reported. Methods showing quantitative determination of CBD in pure isolate was also published earlier^25^. Out of all the methods, the HPLC methods were identified as the simplest as it allows the determination of both acidic and neutral forms and even the system does not require expensive MS detectors. Several methods were published that utilises HPLC^26–31^ and most of these methods employ buffer mobile phases and gradient elution techniques^32,33^. Moreover, these methods demonstrate the separation of cannabinoid mixture where it includes several cannabinoids apart from CBD and THC^28,34–37^. The simplest HPLC methods makes use of isocratic elution techniques where the mobile phase consists of only organic modifiers were also reported^38,39^. The hemp industry demands the development of a simple, robust, accurate and efficient analytical methodologies for the quantification of the main constituents such as CBD and THC, in order to keep up with the rapidly changing regulatory requirements of this industry. Hemp oil is currently used to formulate several novel consumer goods and food supplements. Examples of such products include beverage enhancers, chocolates, capsules, topicals, baked goods, and also as non-prescription medicines in the form of tablets, tinctures, capsules, oral drops, and sublingual drops.

Here, we demonstrate the feasibility of reverse phase HPLC (RP-HPLC) method for reliable and fast quantitative determination of CBD and THC in hemp oil products. This method uses isocratic elution that makes the method feasible to perform in any standard HPLC system. Also, the method performs well in ambient temperature conditions and uses a mobile phase of organic modifier only. Specificity, linearity, accuracy, range, precision, and robustness of the analytical method were determined according to assay method validation requirements specified in the International council for harmonization (ICH) quality guideline Q2(R1), “Validation of Analytical Procedures: Test and Methodology” for quantitation of CBD and THC by liquid chromatography^40^.

## Materials and methods

### Equipment

RP-HPLC analysis was performed on a Jasco Extrema (JASCO, Inc., 28600 Mary’s court, Easton, Maryland 21601, USA) connected with an in built autosampler (AS-4150), quaternary pump (PU-4180) with an on-line degasser, column oven (CO-4061), UV/Vis detector (UV-4075) and an interface box LC-NetII/ADC. Chromatographic data was collected and analysed using ChromNAV Ver.2 software. A second HPLC system (Agilent 1100 series) equipped with variable wavelength detector (G1314A VWD), autosampler (G1313A) and isocratic pump system (G1310A). This system automated with a Chemstation (Agilent technologies) software and which was used for intermediate precision (repeatability) testing and data collection. Chromatographic separations were achieved by using SOLAS 100 Å C18 150 mm × 4.6 mm 5 µm column (Glantreo limited, Ireland). Different HPLC columns such as SOLAS ODS C18 150 mm × 4.6 mm 5 µm, SOLAS ODS C18 150 mm × 4.6 mm 3 µm, SOLAS BDS C18 150 mm × 4.6 mm 5 µm, SOLAS BDS C18 150 mm × 4.6 mm 3 µm and EIROSHELL C18 150 mm × 4.6 mm 2.6 µm (Glantreo limited) were used for this method development purpose. Each Chemical and test samples were accurately weighed using an analytical balance (Adventurer Pro AS214, Ohaus corporation, Pine Brook, NJ USA).

### Chemicals and reagents

All reagents and chemicals of HPLC or analytical grade were used, including acetonitrile from Alfa Aesar (Thermo Fisher scientific, Lancashire, United Kingdom) and distilled water from in-house facility. Cannabidiol (CBD) and Δ^9^-Tetrahydrocannabinol (Δ^9^-THC or THC) reference standards were commercially purchased from Cerilliant (Cerilliant Corporation, Round Rock, Texas, United States). Phytocannabinoid mixture 10 was purchased from Cayman chemical (Cayman chemical company, 1180 East Ellsworth Road, Ann Arbor, Michigan 48108 USA). Prism Science LLC, 30403 Kings Valley Dr., Conifer, CO 80433 kindly provided CBD tablets (Santeer LUV 5 mg CBD Tablets-Medium) and placebo tablets for testing. Millex-LCR PTFE 0.45um membrane filters were purchased from Merck Millipore Ltd., Tullagreen, Carrigtwohill, Co. Cork, Ireland.

### Chromatographic conditions used

A mobile phase of acetonitrile and water 75/25 v/v with isocratic flow rate of 1.5 mL/min were used. Column temperature was maintained at 25 °C and detection was made at 214 nm. 10 µL of standard and sample solutions were injected in the HPLC system, where a run time of 10 min for CBD standard and 20 min for system suitability and assay samples were used. For method development, 20 µL of phytocannabinoid mixture 10 working standard solution was injected in the HPLC system.

### Preparation of stock standard solutions

Stock standard solutions of 100 µg/mL CBD and 100 µg/mL THC were prepared by dissolving 1.0 mL of CBD and THC reference standards to a 10 mL of acetonitrile in volumetric flasks separately. 1.0 mL of THC stock standard solution was diluted by a 10 mL of acetonitrile and was used as final standard stock containing 10 µg/mL of THC.

### Method development

To optimise the chromatographic method parameters, working standard solution of 10 µg/mL CBD, prepared by diluting 1.0 mL of CBD stock standard to 10 mL with acetonitrile. A 25 µg/mL phytocannabinoid mixture-10 working standard solution, prepared by diluting 1.0 mL of 250 µg/mL standard solution to a 10 mL with acetonitrile were used. Mobile phase varying from 60/40 v/v to 80/20 v/v acetonitrile/water were considered and flow rate of 1.5 mL/min and 2.0 mL/min were studied. After optimising the mobile phase and flow rate parameters, testing was conducted in different wavelengths—(214 nm, 228 nm, 230 nm, 240 nm, 280 nm, and 305 nm). Chromatographic columns of 150 × 4.6 mm with different particle size and column chemistry were tested. The columns considered were SOLAS C18 5 µm, SOLAS ODS 5 µm and 3 µm, SOLAS BDS 5 µm and 3 µm, and EIROSHELL C18 2.6 µm. The chromatographic column which showed better separation efficiency was chosen for final testing.

### System suitability

A 1.0 mL of CBD and THC stock standard solutions were diluted by 10 mL of acetonitrile in a volumetric flask (standard mixture of concentration 10 µg/mL CBD and 1 µg/mL THC each) and used for assay method. This solution was used for system suitability testing where six injections were made in HPLC and determined the plate count, tailing factor, resolution, and reproducibility (percent RSD of retention time, peak area, and height for six injections).

### Linearity

Assay linearity was demonstrated by preparing five standard solutions within the range of 50–150% of the nominal sample concentration (0.1 mg/mL). Each solution was prepared by serial dilution from a single stock (5–15 µg/mL for CBD and 0.5–1.5 µg/mL for THC) and was injected in triplicate. Linear regression analysis was performed without considering the origin as a data point.

### Specificity

For specificity, prepared un-spiked placebo solutions by weighing placebo equivalent to one placebo blend tablet weight (weighed 20 tablets and calculated the average weight) and transferred into a 50 mL volumetric flask, added 35 mL of acetonitrile, and sonicated for 20 min. Diluted to volume with acetonitrile and mixed well (un-spiked placebo stock). Diluted 1.0 mL of this solution to 10 mL with acetonitrile, mixed well, filtered into auto sampler vial after discarding 2.0 mL of filtrate using 0.45 µm Millex PTFE filter unit. The placebo preparation was injected in duplicate. Prepared two separate spiked solutions containing the active at 100% by preparing un-spiked placebo stock separately and diluted 1.0–10 mL with acetonitrile, added 1.0 mL of CBD stock standard and 1.0 mL of THC final stock standard solutions, mixed well, filtered into auto sampler vial after discarding 2.0 mL of filtrate using 0.45 µm Millex PTFE filter unit. (Spiked concentration: CBD—10 µg/mL and THC—1 µg/mL each). Injected the spiked samples twice to confirm specificity.

### Range

Range for the assay method was demonstrated by analysing placebo solutions spiked in a range between 50 and 150% of the nominal method sample concentration of CBD (10 µg/mL) and THC (1 µg/mL). Three weights were prepared at each of five concentration levels and each solution was analysed in triplicate. Linear regression analysis was performed without considering the origin as a data point.

### Accuracy

Accuracy and recovery of the method for assay was validated by analysing data obtained from spiked placebo solutions during the range portion of the validation. The percent recovery of each individual sample weight and the average at each concentration level was determined.

### Precision

For precision, assay sample solutions are prepared as follows:

Five tablets (minimum) were accurately weighed, the average tablet weight was calculated and then the tablets were grinded into fine powder. A weighed sample equivalent to 5 mg of CBD transferred into a 50 mL volumetric flask, Approximately 35 mL of acetonitrile was added and the resulting solution was sonicated for 15 min with intermitted shaking. The samples were then diluted to volume with acetonitrile and mixed well. 1.0 mL of this solution was diluted to 10 mL with acetonitrile, mixed well and filtered a portion through Millex PTFE 0.45 µm sample filtration kit into HPLC vials after discarding initial 2 mL of sample solution. Following this procedure, the sample was calculated to contain 10 µg/mL CBD. 10 µL of the sample solutions were subsequently injected on to HPLC system.

For method precision (repeatability), six assay samples prepared and percent label claim for CBD and THC were determined for each sample. Intermediate precision of the method was demonstrated by performing the repeatability experiment again with a second analyst. This analyst used different solution preparations, reagents, operating conditions, column, and HPLC systems and tested on different days from the first analyst.

### Robustness

Method robustness was established by variation of chromatographic parameters to indicate the reliability of the proposed analytical method during normal usage. The chromatographic parameters considered were the variation in mobile phase composition, column oven temperature and mobile phase flow rate. In mobile phase composition increased the major component by 5% and 10%, column oven temperature was increased and reduced by 5 °C and flow rate increased and decreased by 10% and 25% from the proposed method condition. System suitability standard solution was injected in duplicate with each parameter change.

## Results and discussion

The assay method was developed and validated according to ICH guidelines with respect to system suitability, linearity, specificity, accuracy, range, precision (repeatability and intermediate precision) and robustness.

### Method development

The focus of our research was to develop a new analytical method for the simultaneous determination of CBD and THC in hemp oil infused products. Moreover, the method proposed here can be utilized in commercial drug testing laboratories, pharmaceutical industries, and research laboratories as a standard quality control procedure. Optimization of chromatographic conditions plays a major role in the accurate detection and quantification of analytes in HPLC techniques. Mobile phases containing different percentages of acetonitrile (60, 70, 75 and 80%, v/v) were examined and the elution time for CBD and resolution between CBD and CBG were noted. During the testing for different mobile phase compositions, a flow rate of 2.0 mL/min and detection wavelength of 214 nm was employed. A detection wavelength of 305 nm was used to identify the CBN in the phytocannabinoid mixture because CBN generates a strong peak at 305 nm. The test results lead us to commend a mobile phase concentration of 75/25 v/v acetonitrile/water. Flow rate of 1.5 mL/min and 2.0 mL/min were compared and finalised the flow rate as 1.5 mL/min where CBD eluted with better efficiency. Different wavelengths (214 nm, 228 nm, 230 nm, 240 nm, 280 nm, and 305 nm) were checked and the peak intensity for CBD was noted. Different chromatographic columns with 150 mm × 4.6 mm column dimensions were tested for their ability to resolve CBD and THC from other cannabinoids. All the columns tested were manufactured by Glantreo limited using their in-house silica technology platform and it includes SOLAS 100 Å C18 5 µm, SOLAS ODS 5 µm and 3 µm, SOLAS BDS 5 µm and 3 µm, and EIROSHELL C18 2.6 µm. All the test results were tabulated and shown in Table 1a–c.

**Table 1:** Method development.

Mobile phase composition (acetonitrile/water v/v)	Flow rate (mL/min)	Retention time (CBD standard) minutes	Number of theoretical plates (N) CBD Standard	Symmetry (CBD Standard)	Resolution CBG/CBD (Cannabinoid mixture 10)	Retention time CBC (cannabinoid mixture 10) minutes^a^		
**(a): Method development—mobile phase and Flow rate optimization at 214 nm**								
60/40	2.0	18.576	6894	1.09	CBG peak not detected	55.625		
70/30	2.0	7.735	6704	1.14	0.76	21.123		
70/30	1.5	10.292	7995	1.11	0.93	28.018		
75/25	2.0	5.209	6317	1.21	1.12	13.372		
	1.5	6.918	7644	1.19	1.23	17.546		
80/20	2.0	3.654	5648	1.19	1.25	8.724		
	1.5	4.848	6960	1.23	1.39	11.566		

UV wavelength (nm)	Peak area (CBD standard)	Peak height (CBD standard)	N (CBD standard)	Columns used (150 mm × 4.6 mm 100 Å)	Retention time (CBD standard) minutes	N (CBD standard)	Symmetry (CBD standard)	Resolution (CBG/CBD) cannabinoid mixture 10
**(b): Method development—optimization for detector wavelength where mobile phase was fixed as 75/25 v/v acetonitrile/water and flow rate 1.5 mL/min**				**(c): Method development—chromatographic column selection**				
214	374,090	30,108	7644	SOLAS C18 5 µm	6.918	7644	1.191	1.23
228	136,453	10,892	7441	SOLAS ODS C18 5 µm	3.129	3570	1.174	NR
230	122,916	9842	7398	SOLAS ODS C18 3 µm	2.625	6819	1.009	NR
240	46,056	3696	7385	SOLAS BDS C18 5 µm	2.841	6438	1.047	NR
280	10,291	822	7324	SOLAS BDS C18 3 µm	2.863	7481	1.081	NR
305^b^	ND	ND	ND	EIROSHELL C18 2.6 µm	3.123	4278	1.169	0.95

*ND* Not detected, *NR* CBG not resolved from CBD.

^a^CBC was eluted as the last peak in phytocannabinoid mixture 10 and was considered to choose the total run time for the samples.

^b^305 nm was used for identifying the cannabinoid peaks CBN and CBC.

From Table 1a, it is clear that a mobile phase of 75/25 v/v acetonitrile/water with a flow rate 1.5 mL/min enables the CBD peak to elute with better efficiency and also, with comparatively better separation from CBG. Table 1b showed that detector wavelength of both 214 nm and 228 nm were suitable because in higher wavelengths a significant reduction in the strength of peak intensity for CBD has been observed. In this method 214 nm was considered as optimal wavelength as in this wavelength CBD elutes with higher peak intensity and better efficiency (Table 1b). Table 1c indicated that SOLAS C18 150 mm × 4.6 mm 5 µm column demonstrated a well separation for phytocannabinoid mixture 10 with a superior resolution between CBG and CBD as compared to the SOLAS ODS 5 µm, BDS 5 µm and EIROSHELL 2.6 µm C18 columns. Furthermore, SOLAS C18 150 mm × 4.6 mm 5 µm column demonstrated higher efficiency in terms of number of theoretical plates for CBD and also, with CBD peak symmetry ≤ 1.5.

### System suitability testing

**Figure 2:**
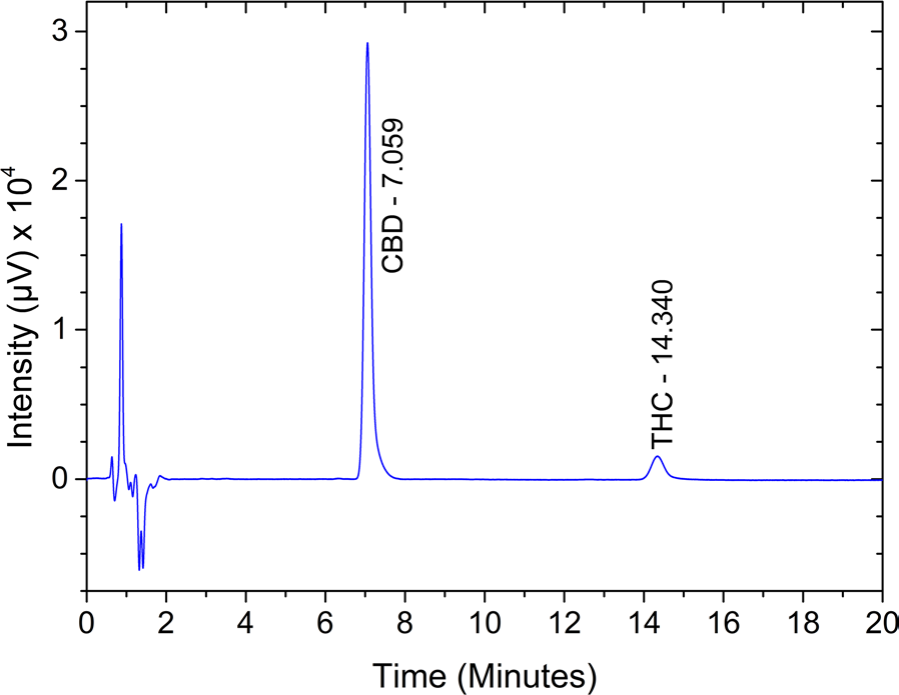
System suitability chromatogram of CBD and THC standard mixture (10 µg/mL CBD and 1 µg/mL THC) for assay.

A system suitability test was performed for standard mixture containing 10 µg/mL CBD and 1 µg/mL THC and six replicate injections were performed in both HPLC systems (Agilent 1100 and Jasco Extrema HPLC’s). The observed RSD values for retention time, peak area, and peak height, are well within the set limits of acceptance criteria of less than 1%. Theoretical plates (N), USP tailing factor (T), Capacity factor, USP resolution (Rs) between CBD and THC, and relative retention time (RRT) of THC were determined for assay. The results are summarized in Table 2a and all results are within the set acceptable limit mentioned in Table 2a. A typical system suitability chromatogram was shown in Fig. 2.

**Table 2:** System suitability and Validation results.

System suitability parameter	Acceptance criteria	Results		Compound	Amount of standard added (µg/mL)	Linearity (r^2^)	Accuracy (% recovery)	Range		Precision (% RSD)		
		CBD	THC					% RSD (3 preparations/9 injections	r^2^	Repeatability	Intermediate precision	%RSD (analyst 1 and 2)
**(a): System suitability parameters for assay method validation**				**(b): Validation results of the RP-HPLC assay test method for CBD and THC**								
Injection precision for retention time (min)	RSD ≤ 1%	0.42	0.53	CBD	5	0.9999	100.14	0.14	0.9997	0.23	0.14	0.26
					7		102.38	0.46				
Injection precision for peak area (n = 6)	RSD ≤ 1%	0.11	0.67		10		105.17	0.62				
Injection precision for peak height	RSD ≤ 1%	0.62	0.68		13		104.86	0.78				
Resolution (Rs)	Rs ≥ 2.0	–	16.12		15		104.38	0.28				
USP tailing factor (T)	T ≤ 2.0	1.35	1.08	THC	0.5	0.9999	100.24	1.22	0.9996	0.43	0.64	0.94
Capacity factor (k)	k ≥ 2.0	10.3	21.8		0.7		98.85	0.65				
Theoretical plates (N)	N ≥ 3000	7970	9504		1.0		98.17	0.84				
Relative retention factor (RRT)	2.0	–	2.03		1.3		98.03	0.64				
					1.5		99.64	0.57

### Linearity

**Figure 3:**
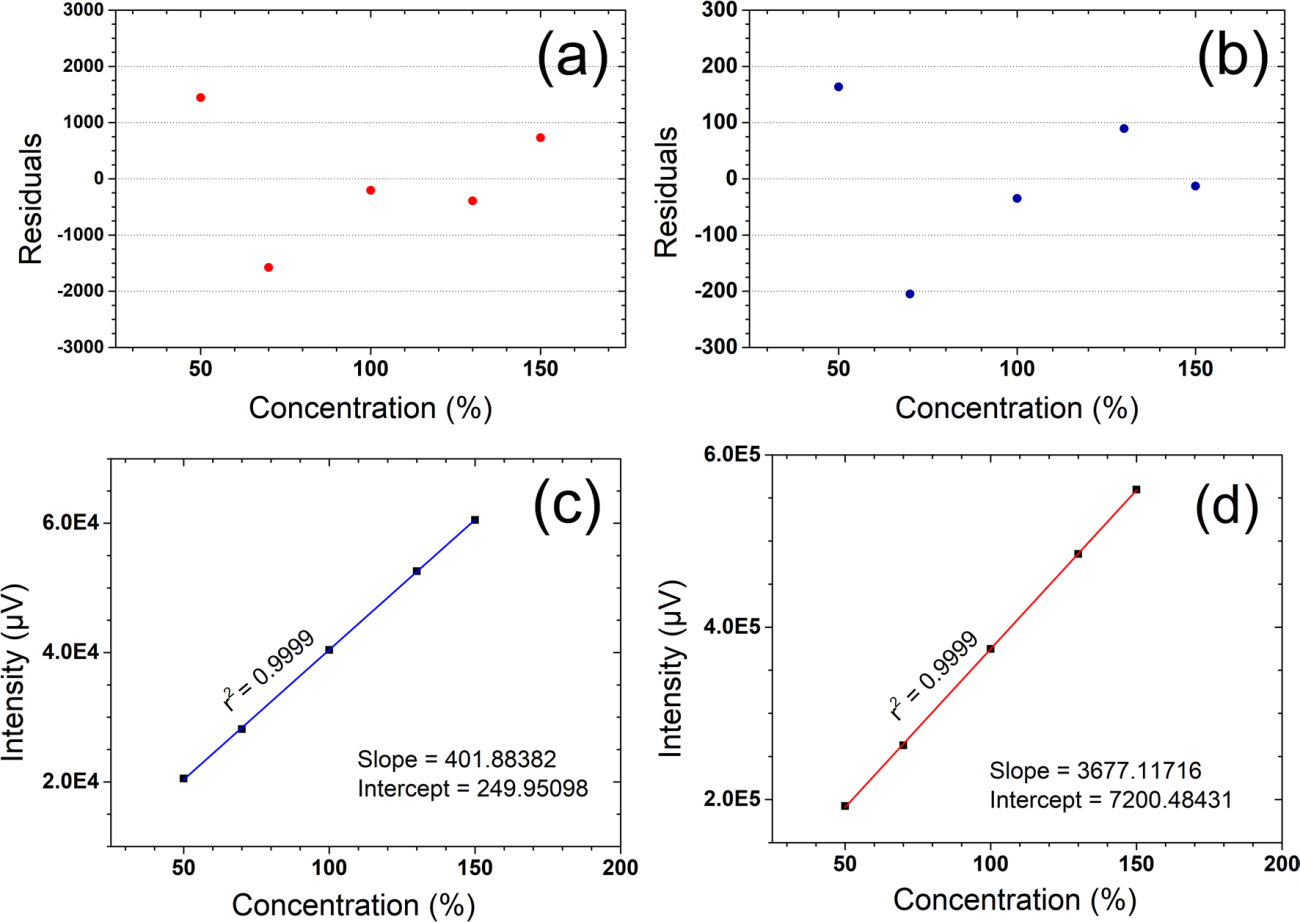
Linearity and residual plot diagrams for CBD and THC assay method validation (**a**) Residual plot for CBD (**b**) Residual plot for THC (**c**) Assay linearity plot for THC and (**d**) Assay linearity plot for CBD.

The linearity of peak area response for CBD and THC were determined for assay method by analysing standard mixture with serial dilutions from 50 to 150% of the method concentration (10 µg/mL for CBD and 1 µg/mL for THC). Linear regression analysis was performed without considering the origin as a data point. For both CBD and THC, percentage of RSD for the peak area for the triplicate injections were found to be less than 1.0% and exhibits linear responses with r^2^ > 0.999 (Table 2b). A graph of concentration versus peak area response (intensity) is shown in Fig. 3. A graph of the residuals versus analyte concentration is shown in Fig. 3.

### Specificity

**Figure 4:**
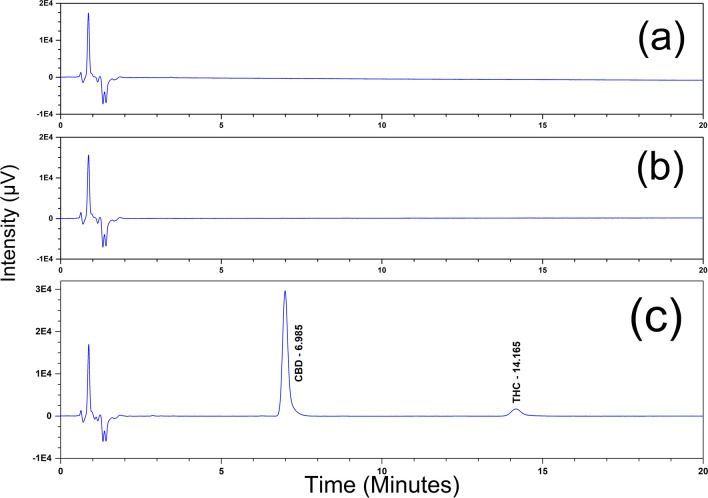
Specificity chromatograms of (**a**) diluent blank (**b**) un-spiked placebo and (**c**) spiked placebo samples of CBD and THC assay.

Placebo samples were prepared by weighing an appropriate amount of placebo (the placebo remains 100% of method concentration), with respect to the concentration specified in the method being validated. The placebo preparation was injected in duplicate. Prepared two separate spiked solutions containing the active at 100%. The spiked samples were injected twice to confirm specificity. Example chromatograms of diluent blank, un-spiked placebo, and spiked placebo preparations are presented in the Fig. 4. Figure 4 demonstrated the selectivity of this method because no interfering peaks from the diluent and placebo were observed. Additionally, a blank sample solvent (acetonitrile) was injected after every 10th sample injection (before the check standard injection) and at the end of each chromatographic sequence in order to evaluate the presence of carry-over since this method follows isocratic elution. These blank sample solvent injections were incorporated in the chromatographic sequence of routine hemp oil infused sample analysis too as a precaution to minimize the effect of accumulated late eluting impurities in the column, if any. The chromatograms for these blank sample solvent injections showed the absence of any peaks related to cannabinoid metabolites or any unknown impurities at the retention times of CBD and THC which in turn proves the absence of on-column accumulation of any unretained compounds.

### Range

**Figure 5:**
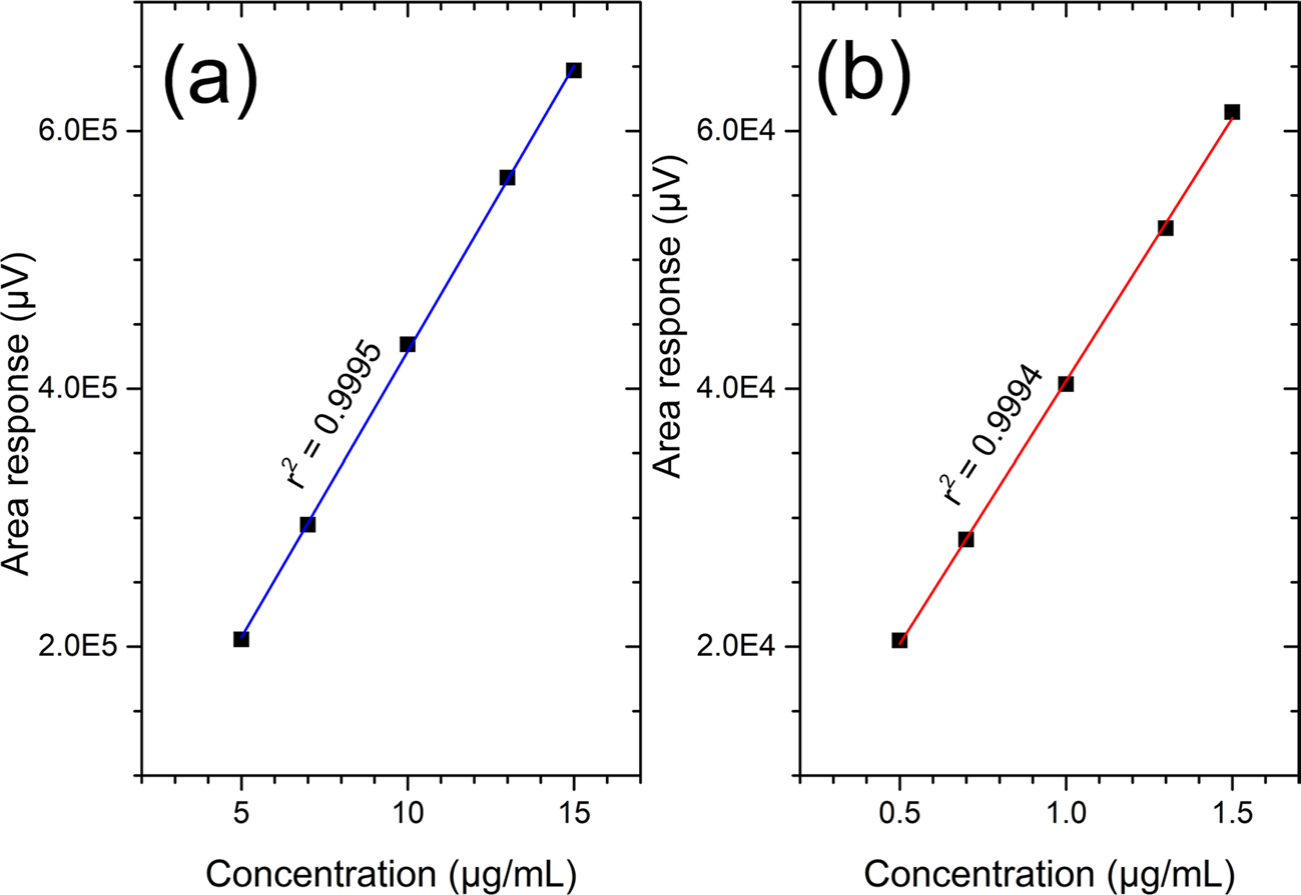
Concentration vs. area response graph of (**a**) CBD and (**b**) THC for accuracy and range testing for assay.

Placebo solutions spiked with sample concentration ranging from 50 to 150% of method concentration was prepared in triplicate. Three injections were made from each preparation, and linear regression analysis was performed without considering the origin as a data point. Both CBD and THC standards show excellent linear response (r^2^ > 0.999 (Table 2b). Also, percentage of RSD for average area responses for the triplicate injections and for each concentration level (n = 9, where n is number of injections made) was found to be less than 2.0% (Table 2b). A graph of concentration versus area response is shown in Fig. 5. A graph of the residuals versus analyte concentration is shown in Fig. 5.

### Accuracy

Percentage recovery at each individual spiked placebo samples and the average at each concentration level was calculated. The results were tabulated in Table 2b. The percentage recovery of the spiked placebos were found to be 100–105% for CBD and 98–100% for THC (acceptance criteria: 90–110% as per ICH guidelines^40^) for the average of each set of three weights. Each individual sample recovery also found within the range of 90–110%. The recovery data shows the high extraction efficiency for CBD and THC of this method.

### Precision

**Figure 6:**
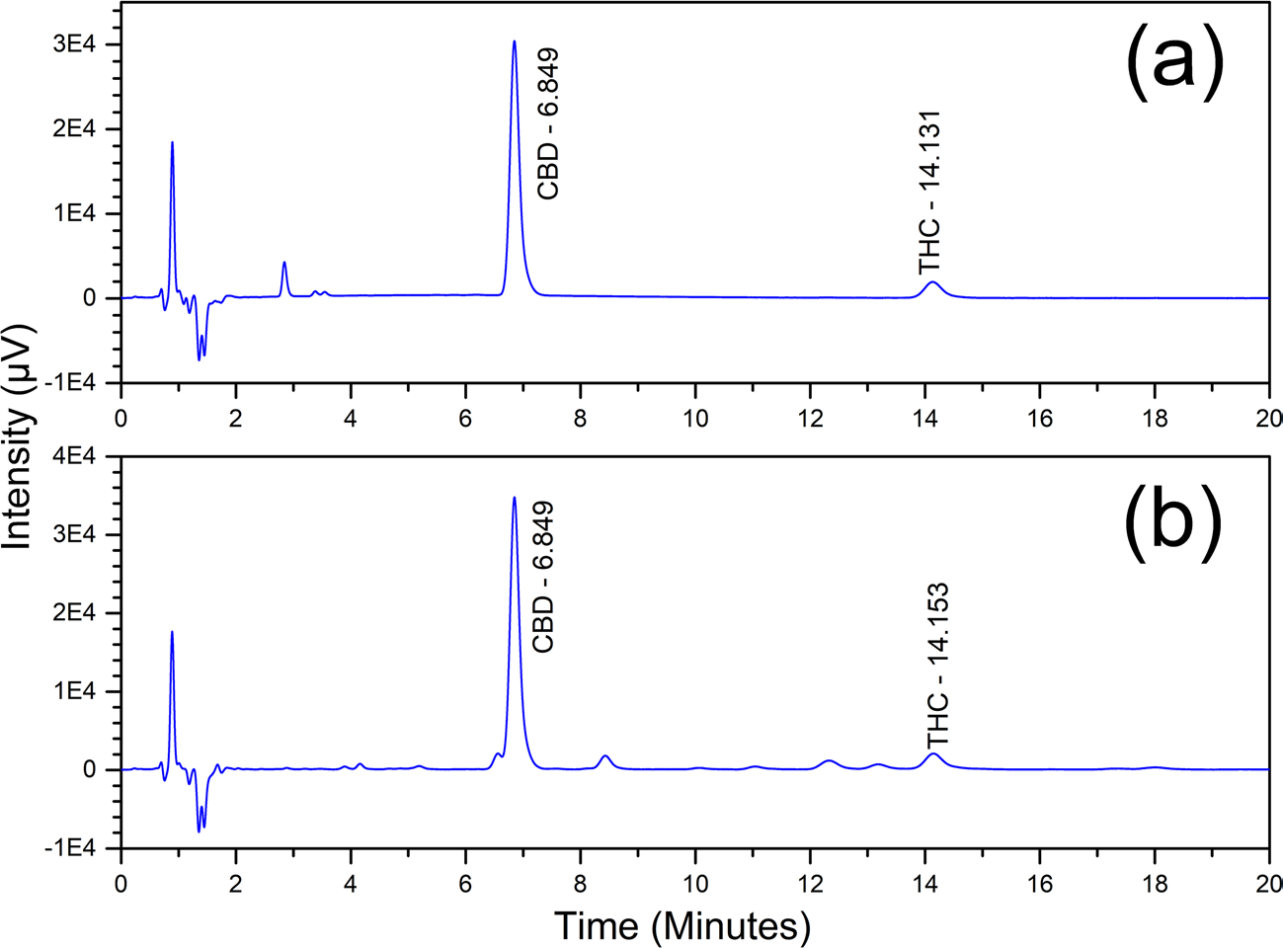
(**a**) Standard and (**b**) sample chromatograms from precision testing for CBD and THC assay method validation.

Method precision (repeatability) was performed by preparing six sample solutions from the tablets for a concentration of 10 µg/mL CBD where equivalent of 5 mg CBD was weighed. Each preparation was injected twice, and the assay values calculated. Results including the percentage RSD values of CBD and THC for both repeatability and intermediate precision are presented in Table 2b. Percentage RSD values for both CBD and THC are below 2.0%. The chromatograms of a typical standard and sample solutions (5 mg) were shown in Fig. 6. The sample chromatogram was compared with the chromatograms of standard, placebo and blank. As CBG was appeared to be slightly overlapping with CBD in the hemp oil sample chromatogram, still the separation was distinct to allow a proper integration. Intermediate precision of the method was demonstrated by repeating the repeatability experiment with a second analyst. This analyst used different solution preparations, reagents, operating conditions, column, and HPLC systems and tested on different days from the first analyst. All acceptance criteria were met (The percentage RSD of the assay values should not be greater than 2.0%. The percentage RSD of the combined assay values from both analysts should be not more than 3.0% as per ICH guidelines).

### Robustness

Method robustness was proved by variation of the mobile phase composition, temperature, and flow rate, from the parameters specified in proposed method. A system suitability standard (10 µg/mL CBD and 1.0 µg/mL THC) was prepared and injected in duplicate with each parameter change. This solution was used to perform robustness for assay and each parameter change was tested one factor at a time. The retention time (RT), theoretical plates (N), Tailing factor (T), Capacity factor (k), resolution between CBD and THC (Rs), and the relative retention time (RRT) for THC are reported in Table 3. Examination of the results from Table 3 suggest that the variation in mobile phase concentration is a critical parameter as with a 5% and 10% decrease in major component (acetonitrile) in volume in the mobile phase composition results elution of THC not within the method run time. Besides, a 5% and 10% increase in major component in mobile phase composition results poor efficiency (reduced theoretical plate values, see Table 3). Variations in column oven temperature shows no variation in any of the system or column efficiency parameters.

**Table 3:** Robustness test for HPLC parameter variations—CBD and THC assay method validation.

Parameter change	CBD				THC				Resolution (CBD/THC)	RRT (THC)
	RT (min)	N	T	K	RT (min)	N	T	K		
Method Conditions	7.03	6960	1.40	10.2	14.30	7974	1.22	21.8	14.90	2.03
Flow − 25%	9.44	7223	1.45	13.2	19.12	7756	1.28	27.8	14.78	2.02
Flow − 10%	7.82	6950	1.43	12.7	15.83	7707	1.19	26.8	14.64	2.02
Flow + 10%	6.38	6361	1.42	10.2	12.91	7243	1.26	21.6	14.14	2.02
Flow + 25%	5.59	6228	1.42	10.0	11.33	7316	1.28	21.2	14.14	2.03
Column temp: + 5 °C	6.92	6864	1.37	10.2	13.91	7711	1.18	21.4	14.48	2.01
Column temp: − 5 °C	7.12	6796	1.51	10.4	14.57	7850	1.32	22.3	14.89	2.05
MP + 10%	4.45	4197	1.29	5.5	8.54	4634	1.26	11.6	10.55	1.92
MP + 5%	5.69	4454	1.33	7.4	11.27	4777	1.20	15.5	11.27	1.98
MP − 5%	9.58	7514	1.36	13.9	19.91	8567	1.07	29.9	15.91	2.08
MP − 10%	12.42	8500	1.25	19.5	25.97	9849	1.02	41.8	17.13	2.09

Additionally, solution stability was established for 48 h by testing system suitability of standard solutions twice by using a freshly prepared standard, the solutions kept in room temperature for 24 h, the solutions kept in 0–8 °C for 24 h and the solution kept in room temperature for 48 h. The results show negligibly small variation in peak area absorbance values and proved the standard solution stability of 48 h in room temperature. Also, an old column where > 500 injections made was tested and found a higher tailing and backpressure as compared to new column indicating approximate lifetime of this HPLC column (SOLAS 100 Å C18, 150 mm × 4.6 mm 5 µm) is less than 500 injections.

### Application of final method to marketed hemp oil infused products

The suitability of this validated method was studied using a wide range of hemp oil infused tablets provided by Prism Science LLC, 30403 Kings Valley Dr., Conifer, CO 80433. The samples included Santeer LUV 2.5 mg, 5 mg, and 10 mg PET tablets, Santeer FOCUS and RENEW 5 mg CBD tablets, and Santeer DREAM 10 mg CBD tablets. Then, this method was applied to quantitate CBD and THC in various hemp oil infused products and presented in a paper published by Analakkattillam et al.^41^. The samples tested consisted of tablets, capsules, tincture, oral drops, beverage-enhancers, and plant extract oils. Furthermore, this method was found to be suitable to quantitate CBD and THC in complex matrices such as in hemp oil infused chocolates where the cannabinoids were extracted using QuEChERS extraction method^42,43^. Furthermore, Glantreo limited presently employs this assay method for testing commercial hemp oil infused samples as a quality control procedure.

Suitability of this method’s efficiency to quantitate CBD and THC was compared by considering the recovery data for CBD and THC reported in different published papers where HPLC technique employed (Supplementary Table S1). The Supplementary Table S1 clearly showed that 10 out of 17 methods employed gradient elution^4,26,28–32,35–37^ and for the remaining 7 methods showed isocratic elution, four methods use an acid or base or salt containing mobile phase^16,27,33,34^. One isocratic elution method utilizes a column oven temperature of 40 °C and was originally an Ultra high performance liquid chromatographic method^25^ whereas in the other isocratic method, a column oven temperature of 55 °C and an autosampler thermostat of 4 °C was employed^39^. The only method that found to be simple used 85% methanol as mobile phase and with reduced runtime of 10 min, but the method showed CBD recovery of 97.1% and THC recovery of 99.3% whereas our method showed a 105.2% recovery for CBD and 100.2% recovery for THC^41^. Moreover, while performing the assay testing for the samples mentioned in the published paper^41^, initially acetonitrile was used as extraction solvent. For the samples where the CBD content was found to be very low as compared to its label claim, conducted the analysis using methanol as extraction solvent. For the samples where the CBD was not detected, further performed extraction using QuEChERS extraction method^42,43^ in order to confirm the extraction efficiency of acetonitrile and/or methanol. The assay test results were comparable for both acetonitrile and methanol extractions where acetonitrile found to be more suitable.

## Conclusion

The precise quantification of compounds like cannabinoids where the products contain psychotic molecules such as THC, demands that laboratories have affordable and easily applicable validated procedures. The amount of THC within a product determines the legal restriction for the said product. The assay test method validated and presented here allows the quantitative and simultaneous determination of CBD and THC in Hemp oil infused products in 20 min run time. The method uses single wavelength of 214 nm, and the testing can be carried out using any traditional standard RP-HPLC and UV–visible detectors. Moreover, the method utilizes only organic solvents for standard and preparation, and for mobile phase single organic modifier was involved with no pH adjustment and the testing follows an isocratic elution. Due to simplicity and consistency this method can be employed by any analyst with basic HPLC knowledge. The method was shown to be selective, precise, linear, and accurate within the range of 50% to 150% of the nominal concentration for tablet strengths. The method was shown to be robust with the established as critical parameters such as mobile phase concentration changes and flow rate.

## Supplementary Information

Supplementary Information.

## Data Availability

The authors confirm that the data supporting the results of this study are available upon request from the corresponding author Dr. Eric Moore (e.moore@ucc.ie).

## Abbreviations

RP-HPLC: Reverse phase high performance liquid chromatography
CBD: Cannabidiol
THC: Tetrahydrocannabinol
RSD: Relative standard deviation
QC: Quality control
ICH: International council for harmonisation
USP: United States pharmacopeia

## References

[1] ElSohly MA, Mehmedic Z, Foster S, Gon C, Chandra S, Church JC (2016). Changes in cannabis potency over the last 2 decades (1995–2014): Analysis of current data in the United States. Biol. Psychiatry..

[2] ElSohly MA, Radwan MM, Gul W, Chandra S, Galal A (2017). Phytochemistry of *Cannabis sativa* L. Phytocannabinoids.

[3] Radwan, M. M., Wanas, A. S., Chandra, S. & ElSohly, M. A. Natural cannabinoids of cannabis and methods of analysis. In *Cannabis sativa L.—Botany and Biotechnology* (eds. Chandra, S., Lata, H., ElSohly, M. A.) 161–182 (Springer International Publishing, 2017). 10.1007/978-3-319-54564-6_7.

[4] Zivovinovic S, Alder R, Allenspach MD, Steuer C (2018). Determination of cannabinoids in *Cannabis sativa* L. samples for recreational, medical, and forensic purposes by reversed-phase liquid chromatography-ultraviolet detection. J. Anal. Sci. Technol..

[5] Mechoulam R (2005). Cannabinoids as Therapeutics.

[6] U.S.C. United States Code. 2015 Edition. Title 7—Agriculture, Chapter 88—Research, Subchapter VII—Miscellaneous Research Provisions, Sec. 5940—Legitimacy of industrial hemp research. In *U.S.G.P. Office, United States*. 1688 (ed. U.S.G.P. Office) (2015).

[7] Villamor JL, Bermejo AM, Tabernero MJ, Fernández P (2004). Determination of cannabinoids in human hair by GC/MS. Anal. Lett..

[8] Santos NA (2019). Analysis of isomeric cannabinoid standards and cannabis products by UPLC-ESI-TWIM-MS: A comparison with GC-MS and GC× GC-QMS. J. Braz. Chem. Soc..

[9] Lachenmeier DW, Kroener L, Musshoff F, Madea B (2004). Determination of cannabinoids in hemp food products by use of headspace solid-phase microextraction and gas chromatography-mass spectrometry. Anal. Bioanal. Chem..

[10] Rodrigues A, Yegles M, Van Elsué N, Schneider S (2018). Determination of cannabinoids in hair of CBD rich extracts consumers using gas chromatography with tandem mass spectrometry (GC/MS–MS). Forensic Sci. Int..

[11] Cardenia V, Gallina Toschi T, Scappini S, Rubino RC, Rodriguez-Estrada MT (2018). Development and validation of a Fast gas chromatography/mass spectrometry method for the determination of cannabinoids in *Cannabis sativa* L. J. Food Drug Anal..

[12] Leghissa A, Hildenbrand ZL, Foss FW, Schug KA (2018). Determination of cannabinoids from a surrogate hops matrix using multiple reaction monitoring gas chromatography with triple quadrupole mass spectrometry. J. Sep. Sci..

[13] Hillig KW, Mahlberg PG (2004). A chemotaxonomic analysis of cannabinoid variation in Cannabis (Cannabaceae). Am. J. Bot..

[14] Karschner EL, Barnes AJ, Lowe RH, Scheidweiler KB, Huestis MA (2010). Validation of a two-dimensional gas chromatography mass spectrometry method for the simultaneous quantification of cannabidiol, Δ9-tetrahydrocannabinol (THC), 11-hydroxy-THC, and 11-nor-9-carboxy-THC in plasma. Anal. Bioanal. Chem..

[15] Dussy FE, Hamberg C, Luginbühl M, Schwerzmann T, Briellmann TA (2005). Isolation of Δ9-THCA-A from hemp and analytical aspects concerning the determination of Δ9-THC in cannabis products. Forensic Sci. Int..

[16] Deidda R (2019). Analytical quality by design: Development and control strategy for a LC method to evaluate the cannabinoids content in cannabis olive oil extracts. J. Pharm. Biomed. Anal..

[17] Sherma J, Rabel F (2019). Thin layer chromatography in the analysis of cannabis and its components and synthetic cannabinoids. J. Liq. Chromatogr. Relat. Technol..

[18] Aizpurua-Olaizola O, Omar J, Navarro P, Olivares M, Etxebarria N, Usobiaga A (2014). Identification and quantification of cannabinoids in *Cannabis sativa* L. plants by high performance liquid chromatography-mass spectrometry. Anal. Bioanal. Chem..

[19] Isaac G (2017). Ultra-high performance supercritical fluid chromatography applications for natural products analysis. Planta Med. Int. Open..

[20] Lebel P, Waldron KC, Furtos A (2015). Rapid determination of 24 synthetic and natural cannabinoids for LC-MS-MS screening in natural products and drug inspection applications. Curr. Trends Mass Spectrom. Suppl. LCGC N. Am..

[21] Quintela, O. & Crouch, D. J. The determination of cannabinoids using liquid chromatography with mass spectrometric detection. In *LC-MS in Drug Analysis. Methods Mol. Biol. (Methods and Protocols)*, vol. 902 (eds. Langman, L. & Snozek, C.) (Humana Press, 2012) 75–90. 10.1007/978-1-61779-934-1_7. 10.1007/978-1-61779-934-1_7 22767109

[22] Schwope DM, Scheidweiler KB, Huestis MA (2011). Direct quantification of cannabinoids and cannabinoid glucuronides in whole blood by liquid chromatography–tandem mass spectrometry. Anal. Bioanal. Chem..

[23] McRae G, Melanson JE (2020). Quantitative determination and validation of 17 cannabinoids in cannabis and hemp using liquid chromatography-tandem mass spectrometry. Anal. Bioanal. Chem..

[24] Hazekamp, A. *A general introduction to cannabis as medicine. In: Cannabis: extracting the medicine.* PhD diss., Institute of Biology Leiden (IBL), Faculty of Science, Leiden University (2007).

[25] Layton CE, Aubin AJ (2018). Method validation for assay determination of cannabidiol isolates. J. Liq. Chromatogr. Relat. Technol..

[26] De Backer B (2009). Innovative development and validation of an HPLC/DAD method for the qualitative and quantitative determination of major cannabinoids in cannabis plant material. J. Chromatogr. B..

[27] Ambach L, Penitschka F, Broillet A, König S, Weinmann W, Bernhard W (2014). Simultaneous quantification of delta-9-THC, THC-acid A, CBN and CBD in seized drugs using HPLC-DAD. Forensic Sci. Int..

[28] Patel B, Wene D, Fan ZT (2017). Qualitative and quantitative measurement of cannabinoids in cannabis using modified HPLC/DAD method. J. Pharm. Biomed. Anal..

[29] Fekete S (2018). Implementation of a generic liquid chromatographic method development workflow: Application to the analysis of phytocannabinoids and *Cannabis sativa* extracts. J. Pharm. Biomed. Anal..

[30] Citti C, Pacchetti B, Vandelli MA, Forni F, Cannazza G (2018). Analysis of cannabinoids in commercial hemp seed oil and decarboxylation kinetics studies of cannabidiolic acid (CBDA). J. Pharm. Biomed. Anal..

[31] Mudge EM, Murch SJ, Brown PN (2017). Leaner and greener analysis of cannabinoids. Anal. Bioanal. Chem..

[32] Layton, C. & Reuter, W. M. Analysis of Cannabinoids in Hemp Seed Oils by HPLC using PDA detection. Application Note. PerkinElmer, Shelton, CT, Unites States. (2015). https://cdn.technologynetworks.com/tn/Resources/pdf/analysis-of-cannabinoids-in-hemp-seed-oils-by-hplc-using-pda-detection.pdf. (accessed 13 May 2019).

[33] De Vita D (2020). Comparison of different methods for the extraction of cannabinoids from cannabis. Nat. Prod. Res..

[34] Aubin, A. J., Layton, C. & Helmueller, S. Separation of 16 cannabinoids in cannabis flower and extracts using a reversed phase isocratic HPLC method, waters application note 720006426EN (2018).

[35] Pellati F, Brighenti V, Sperlea J, Marchetti L, Bertelli D, Benvenuti S (2018). New methods for the comprehensive analysis of bioactive compounds in *Cannabis sativa* L. (hemp). Molecules.

[36] Lehmann T, Brenneisen R (1995). High performance liquid chromatographic profiling of cannabis products. J. Liq. Chromatogr..

[37] Mandrioli M, Tura M, Scotti S, Gallina Toschi T (2019). Fast detection of 10 cannabinoids by RP-HPLC-UV method in *Cannabis sativa* L. Molecules.

[38] Saingam W, Sakunpak A (2018). Development and validation of reverse phase high performance liquid chromatography method for the determination of delta-9-tetrahydrocannabinol and cannabidiol in oromucosal spray from cannabis extract. Rev. Bras. Farmacogn..

[39] Zgair A (2015). Development of a simple and sensitive HPLC-UV method for the simultaneous determination of cannabidiol and Δ(9)-tetrahydrocannabinol in rat plasma. J. Pharm. Biomed. Anal..

[40] Guideline I. H. T. Validation of analytical procedures: Text and methodology Q2 (R1) version 4. In *Proceedings of the International Conference for Harmonization; Geneva, Switzerland* (2005).

[41] Analakkattillam S, Langsi VK, Hanrahan JP, Moore E (2021). Comparative study of Dissolution for Cannabidiol in EU and US Hemp oil products by HPLC. J. Pharm. Sci..

[42] Raynie DE (2018). General applications of QuEChERS extraction in the isolation of cannabinoids. Cannabis Sci. Technol..

[43] Extraction of Cannabinoids in Marijuana and Edibles by QuEChERS, Application Notes & Papers. United Chemical Technologies, 2731 Bartram Road, Bristol, PA19007. (2016). https://www.unitedchem.com/wp-content/uploads/2019/08/5102-02_Cannabinoids_in_Marijuana_and_Edibles_by_QuEChERS.pdf. (accessed 18 Apr 2022).

