# Supplementary material for: Analytical method validation for assay determination of cannabidiol and tetrahydrocannabinol in hemp oil infused products by RP-HPLC
Source: Sci Rep. 2022 Jul 21;12:12453. doi: 10.1038/s41598-022-13737-6 (PMC9304360; doi:10.1038/s41598-022-13737-6)
Supplement: Supplementary file 1 — Supplementary Information. [file 41598_2022_13737_MOESM1_ESM.pdf]

Title: Analytical method validation for assay determination of cannabidiol and tetrahydrocannabinol in hemp oil infused products by RP-HPLC

Sandhyarani Analakkattillam[1,2], Victor K. Langsi[1], John P. Hanrahan[1] and Eric Moore[2*]

[1] *Glantreo Limited, ERI Building, Lee Road, Cork City, Ireland*

[2] *School of Chemistry, University College Cork, Cork, Ireland*

Supplementary Table S1: Compilation of Chromatographic conditions and percentage recovery for CBD and THC in various published methods used HPLC technique.

Supplementary Table S1

Ref. No. (References)	Author details and DOI	Chromatographic conditions							Study samples used	Percentage Recovery		Remarks
		MP	Elution Mode	Flow rate (mL/minutes)	Detection Wavelength	Column Oven (°C)	Total runtime (minutes)	Column dimensions		CBD	THC or Δ8-THC or Δ9-THC	
4	Zivovinovic, S., Alder, R., Allenspach, M. D. & Steuer, C. 10.1186/s40543-018-0159-8	(A) 0.1% Formic acid in water (B) 0.1% Formic acid in Acetonitrile	Gradient	0.8	220 nm	50	20	150 x 4.6 mm 2.6µm (Phenomenex Kinetex XB-C18)	CBD-hemp tobacco samples and Forensic cannabis sample	106.8%	102.7%	
16	Deidda, R. et al. 10.1016/j.jpba.2019.01.032	75/25 Acetonitrile/ water containing 5 mM of K2HPO4 adjusted to pH 3.45 v/v	Isocratic	0.38	222 nm	53	6	150 x 2.1 mm 2.7µm column & 5 x 2.1 mm 2.7µm Guard column (Agilent Poroshell 120 SB-C18)	Cannabis Olive oil extracts	98.4% – 101.4 %	99.2% - 101.6%	
25	Layton, C. E. & Aubin, A. J. 10.1080/10826076.2018.1424637	45% Acetonitrile in water	Isocratic	0.75	228 nm	40	5	75 x 2.1 mm 1.7µm (ACQUITY UPLC CSH Fluoro-Phenyl)	CBD Isolates	99% - 102%	Not reported	UPLC method and not transferred to HPLC

26	De Backer, B. et al. 10.1016/j.jchromb.2009.11.004	Methanol/50 mM ammonium formate buffer pH 5.19	Gradient	0.3	200 – 400nm	30	36	250 x 2.1 mm 5µm column & 10 x 2.1 mm 5µm guard column (Waters XTerra MS C18)	Cannabis plant material	109.1 ± 2.3 %	104.0 ± 2.1 %	
27	Ambach, L. et al. 10.1016/j.forsciint.2014.06.008	64/36 Acetonitrile/ 25 mM triethylammonium phosphate buffer	Isocratic	1.0	210 nm	Not mentioned	18	LiChroCart 125-4, 5mm with LiChrospher 60, RP-Select B, 5µm Pre-column	Seized cannabis product (cannabis plant and oil samples)	Not reported	Not reported	
28	Patel, B., Wene, D. & Fan, Z. T. 10.1016/j.jpba.2017.07.021	(A) 25 mM ammonium acetate pH 4.75 adjusted with acetic acid. (B) Methanol	Gradient	0.70	235 nm	30	10	75 x 3.0 mm 2.7µm (Agilent Poroshell 120 SB-C18)	Cannabis flower samples	87.6% - 107.0%	81.2% - 102.7%	
29	Fekete, S. et al. 10.1016/j.jpba.2018.03.059	(A) 20 mM ammonium acetate pH 5.8 (B) Acetonitrile	Gradient	0.57	220 nm	50	12	100 x 2.1 mm 1.7µm (BEH Shield RP18)	Cannabis sativa colas	Not reported	Not reported	Focused on Method optimization and not validation
30	Citti, C. et al. 10.1016/j.jpba.2017.11.044	(A) 0.1% Formic acid in water (B) 0.1% Formic acid in acetonitrile	Gradient	0.4	228 nm	25	15	100 x 3.0 mm 2.7µm (Agilent Poroshell 120 EC-C18)	Hemp seed oils	89.7% - 99.6%	91.0 % - 99.6%	

31	Mudge, E. M., Murch, S. J. & Brown, P. N. 10.1007/s00216-017-0256-3	(A) 10 mM ammonium formate, pH 3.6<br>(B) Acetonitrile	Gradient	0.6	220 nm	Not mentioned	15 minutes and 7 minutes equilibration	100 x 3.0 mm 1.7µm (Phenomenex Kinetex C18)	Cannabis flowers and oils	91.3% - 95.5%	90.7% - 99.2%	Autosampler at 4°C
32	Layton, C. & Reuter, W. M. PerkinElmer Application Note	(A) 0.1% Formic acid in water<br>(B) 0.1% Formic acid in acetonitrile	Gradient	1.0	210 nm	50	8 minutes and 7 minutes equilibration	150 x 4.6 mm 3.0µm (PerkinElmer Brownlee Analytical C18)	Hemp seed oil	91.0% - 95.6%	96.6% - 110.2%	
33	De Vita, D. et al. 10.1080/14786419.2019.1601194	80/20 Acetonitrile/ water (0.1% Trifluoracetic acid) v/v	Isocratic	1.0	220 nm	30	11	150 x 4.6 mm 3.5µm (Waters Symmetry C18)	Industrial hemp and medicinal cannabis extracts	Not reported	Not reported	This article focused on extraction techniques, hence full validation testing not conducted.

34	Aubin, A. J., Layton, C. & Helmueller, S. Waters Application Note	59/41 Acetonitrile/ 0.1% Trifluoroacetic acid in water v/v	Isocratic	2.0	228 nm	35	26	150 x 4.6 mm 2.7µm (Cortecs Shield RP18)	Cannabis plant materials and extracts	Not reported	Not reported	This application note, only linearity test performed, and no other validation parameters studied.
35	Pellati, F. et al. 10.3390/molecules23102639	(A) 0.1% Formic acid in water.<br>(B) 0.1% Formic acid in acetonitrile	Gradient	0.4	210 and 220 nm	30	22 minutes and 15 minutes equilibration	150 x 3.0 mm 2.7µm (Ascentis Express C18)	Hemp inflorescences	90.84 ± 0.60	Not reported	
36	Lehmann, T. & Brenneisen, R. 10.1080/10826079508009265	(A) 8.64 g/L orthophosphoric Acid (85%) in water<br>(B) Acetonitrile	Gradient	0.2	210 and 224 nm	40	60	200 x 2.0 mm column with a 20 x 2.0 mm guard column (Spherisorb ODS-1, 3µm)	Herbal cannabis, cannabis resin or cannabis oil	Not reported	Not reported	

37	Mandrioli, M. et al. 10.3390/molecules24112113	(A) 0.085% phosphoric acid in water (B) 0.085% phosphoric acid in acetonitrile.	Gradient	1.6	220 nm	35	10	150 × 4.6 mm 2.7µm with 5 × 4.6 mm 2.7µm guard column (Nex-Leaf CBX Potency)	Cannabis sativa L. inflorescences	84.92%	99.7% - 100%	Autosampler at 4°C
38	Saingam, W. & Sakunpak, A. 10.1016/j.bjp.2018.08.001	85/15 Methanol/Water v/v	Isocratic	1.0	220 nm	25	10	100 x 4.6 mm 3.5µm (ZorbaxC-18)	Cannabis extract and oromucosal spray formulation.	95.6% - 97.1%	97.3% – 99.3%	
39	Zgair, A. et al	62/38 acetonitrile/ water v/v	Isocratic	1.0	220 nm	55	20	150 x 4.6 mm 3µm column with 3µm guard cartridge (ACE C18-PFP)	Rat plasma	86.2% - 91.0%	86.7% - 94.6%	Autosampler at 4°C
